# Dietary fiber and risk of irritable bowel syndrome: a case-control study 

**Published:** 2018

**Authors:** Fatemeh Hosseini Oskouie, Homayoun Vahedi, Mohammad Amin Shahrbaf, Amir Sadeghi, Bahram Rashidkhani, Azita Hekmatdoost

**Affiliations:** 1 *Department of Clinical Nutrition and Dietetics, Faculty of Nutrition and Food Technology, National Nutrition and Food Technology Research Institute, Shahid Beheshti University of Medical Sciences, Tehran, Iran*; 2 *Digestive Disease Research Center, Digestive Disease Research Institute, Tehran, Iran*; 3 *Gastroenterology and Liver Diseases Research Center, Research Institute for Gastroenterology and Liver Diseases, Shahid Beheshti University of Medical Sciences, Tehran, Iran *

**Keywords:** Irritable bowel syndrome, Dietary fiber, IBS, Case-control

## Abstract

**Aim:**

The purpose of this study was to determine the relationship between dietary fiber intake and risk of irritable bowel syndrome (IBS).

**Background:**

Patients with IBS are usually concerned about their diet, which can exacerbate or relieve their symptoms.

**Methods:**

In this case-control study, ninety cases and 355 controls were selected from a gastroenterology clinic. Dietary intakes of participants were assessed using a validated and reliable food frequency questionnaire (FFQ). Dietary fiber was calculated according to United States Department of Agriculture (USDA) food composition table.

**Results:**

Dietary total fiber intake was significantly associated with lower risk of IBS. The adjusted odds ratio (OR) comparing the highest tertile of dietary total fiber with the lowest tertile was 0.14 (95% CI = 0.71–0.28; P-test for trend <0.001); however, there was no significant association or dose–response trend for higher intakes of soluble, and insoluble fiber separately with risk of IBS.

**Conclusion:**

Our data indicate that dietary fiber is inversely associated with the risk of IBS. Further prospective studies are needed to confirm these data.

## Introduction

 Irritable bowel syndrome (IBS) is a functional disorder characterized by frequent abdominal pain, and changes in bowel habits without any pathologic finding in gastrointestinal evaluation (1-3). Possible risk factors for this syndrome include young age, female gender, stress, and depression; however, the role of smoking as a risk factor is controversial in various studies (4).

Although the pathogenesis of IBS has not yet been elucidated, it seems that it is a multifactorial syndrome, with several mechanisms such as micro-inflammation, changes in gut microbiota, high visceral sensitivity, and immune dysfunction (5). Diet plays a pivotal role in aggravation and amelioration of symptoms in patients with IBS. More than 60% of IBS patients report that their symptoms begin or worsen 15 minutes to 3 hours after eating. Although the role of food in relation to gastrointestinal problems is well known, there is no consensus on food items, which can worsen or relieve symptoms of the syndrome (6).

Low FODMAPs (fermentable oligo- di- mono- saccharides and polyoles) diet is considered as the first approach for management of IBS (7). The main sources of dietary fiber such as several fruits and vegetables, legumes, and grains should be avoided in low FODMAPs diet, while some studies have shown beneficial effects of dietary soluble fiber in amelioration of IBS symptoms (8-14). Due to these controversies, we designed the current study to assess the relationship between dietary fiber intakes and risk of IBS. 

## Methods

This case-control study was approved by the Ethics Committee of the National Nutrition and Food Technology Research Institute (NNFTRI), with the ethics number of NNFTRI206. Study participants included ninety patients with IBS, and 355 healthy people selected by simple sampling method among the patients and their accompanied individuals referring to a gastroenterology clinic in Tehran from May 2017 to January 2018. Case and control groups were matched based on their age (5 years) and sex. Cases were newly diagnosed patients with IBS, and diagnosis was confirmed by a gastroenterologist within last month. Controls were asked about the ROME-IV criteria, and included in the study if they did not fulfill the criteria.

All participants were interviewed by an expert nutritionist, and data on age, sex, smoking, alcohol consumption, taking non-steroidal anti-inflammatory medications, oral contraceptives, antibiotics, degree of education, family history of IBS were obtained by completing the general information questionnaire and information about their dietary intakes were obtained by completing a 168-item valid and reliable food frequency questionnaire (FFQ) (15).

During the interview, the average size of each of the food items in the FFQ was explained to participants, and then they were asked about the frequency of consuming any of the food items in the questionnaire during the past year before detecting IBS for patients and one year before interview in control group.

Data obtained through FFQ was analyzed using Nutritionist 4 software and the daily intake of each individual in terms of fiber types was determined. Weight was measured in light weight clothing with a precision of 100 grams, and height was measured without shoes, by meters installed on the wall with a precision of 0.5 cm.

Data were analyzed using SPSS software version 22. Chi-Square test and Fisher test were used to compare the qualitative confounding variables between the case and control groups. In addition, T-test or Mann-Whitney tests were used to compare the confounding variables between the case and control groups; we also calculated odds ratios (OR) of each independent variable on the development of IBS, using logistic regression analysis. 

## Results

Out of 90 cases 59 (65.6%) were female and 31 (41.1%) were men. Of the 355 controls, 197 were female (55.4%) (Table 1). As shown in Table 2, the anthropometric indices were similar in both groups. Of the 90 patients, 33 patients (37%) had constipation dominant IBS, of which 24 were female and 9 were men with an average age of 45. Thirty seven subjects (41.1%) had IBS mixed diarrhea, constipation of which 26 were women and 11 men with an average age of 41, and 20 patients had diarrhea dominant IBS, of which 9 were female and 11 were men with an average age of 35.

**Table 1:** Demographic characteristics of the study participants

Variable	Patients (%)	Control (%)	P value
Number	90	355	
Sex			0.07
Male	(1.41) 31	(.1%45) 158	
Age(year)	41.3±14.2	42.8±12.5	
Education			
Elementary	(.01) 1	(5.4) 15	0.11
Secondary school	(.043) 38	(7.46) 165	
Academic	(7.56) 51	(1.49) 174	
Family history	(2.32) 29	((312	0.04
Current smoker	(9.18) 17	(20) 161	0.15
Consumption of alcohol	(.918) 17	(19) 158	0.95
Total energy intake (kcal)	2758.98 ± 962.59	2806.60 ± 844.75	0.50
Physical activity Low Normal Intense	62(9.68) 23(6.25) 4(4.4)	205(6.57) 114(1.32) 36(3.1)	0.04

Mann-Whitney or T test has been used to compare quantitative variables. - For all quantitative variables, the values ​​are reported as mean±SD, and qualitative variables are expressed as n(%). Chi square or Fisher test was used to compare qualitative variables.

There was no significant relationship between the types of IBS and the mean age of the patients (Table 3). The mean age of the case group was 41.3±14.2 and the mean age of the control group was 43.0±12.2 years. The family history of IBS and alcohol intake in case group was significantly more than the control group and the level of physical activity and smoking in the case group were significantly lower than the control group. No significant relationship was found between education levels and IBS.

**Table 2:** Anthropometric characteristics of the study participants

Variable	Patients (SD)Mean	Control (SD)Mean	P value
Height (m)	(7.9)6 .1	(8.9)1.6	0.77
Weight (kg)	(4.14)71.2	(3.13)73.3	0.35
BMI (kg/m2)	(3.5)8 .27	(27.2 (1.3	0.09

Mann-Whitney or T test has been used to compare quantitative variables. For all quantitative variables, the values ​​are reported as mean±SD, and qualitative variables are expressed as n(%). BMI: Body mass index

**Table 3:** Types of IBS and Mean of year in the case population

Type of IBS	Number (%)	Mean of age (SD)	P value
IBS-C	(7.36) 33	(6.12)45	0.07
IBS-MIX	(1.41) 37	(2.12) 2.41	
IBS-D	(1.22) 20	(1.9)35.2	

One-way Anova analysis was used to compare qualitative variables with mean age. For all quantitative variables, the values ​​are reported as mean±SD, and qualitative variables are expressed as n(%).

IBS-C: Irritable bowel syndrome with constipation; IBS-MIX: Irritable bowel syndrome with constipation and diarrhea; IBS-D: Irritable bowel syndrome with diarrhea

Table 4 shows multivariate adjusted ORs (95% CIs) for the case group according to the tertile of each dietary fiber variable. Dietary total fiber intake was significantly associated with lower risk of IBS. The adjusted odds ratio (OR) comparing the highest tertile of dietary total fiber with the lowest tertile was 0.14 (95% CI = 0.71–0.28; P-test for trend <0.001); however, There was no significant association or dose–response trend for higher intakes of soluble and insoluble fiber separately with risk of IBS.

**Table 4:** Odds ratios and 95% confidence intervals for receiving fiber as risk factors

Variable	Tertile1	Tertile2	Tertile3	P for trend
Total fiber				
Crude	1.00	0.35 (0.21-0.58)	0.13 (0.69-0.28)	<0.001
Adjusted*	1.00	0.32 (0.11-0.54)	0.14 (0.71-0.28)	<0.001
The amount of tertile (gr/d)	<34.5	34.5-48.5	>48.5	
Soluble fiber				
Crude	1.00	1.67 (0.78-3.56)	1.35 (0.63-2.91)	0.18
Adjusted	1.00	1.63 (0.72-3.67)	1.55 (0.69-3.49)	0.24
The amount of tertile (gr/d)	<0.4	0.4-0.6	>0.6	
Insoluble fiber				
Crude	1.00	1.00 (0.46-2.14)	1.42 (0.68-3.00)	0.34
Adjusted	1.00	1.06 (0.47-2.37)	1.41 (0.64-3.12)	0.38
The amount of tertile (gr/d)	<2.2	2.2-3.6	>3.6	

* Data for intake of energy, protein, fat, and carbohydrate, familial history of IBS and intestinal infections, alcohol consumption, smoking and physical activity levels have been adjusted.

## Discussion

To our knowledge, this is the first case-control study that evaluated the association between dietary fiber intake and risk of IBS. Our data have shown that there was a significant correlation between total fiber and risk of IBS; however, there was no significant relationship between soluble and insoluble fiber with IBS.

There are controversies about the association between dietary fiber intake and IBS in previous studies. In a cross-sectional study, Khayatzadeh *et al.* have shown that the Lacto-vegetable dietary pattern containing varieties of vegetables, fruits, and dairy products reduced the symptoms of the syndrome (9). Insoluble fiber increases stool volume and accelerates the intestinal passage by stimulating the intestinal mucosal mechanism to increase their secretion and peristalsis. Various types of fiber like psyllium are fermented quantitatively or absorb water, which normalizes the stool form. Evidence suggest that dietary fiber can act as a prebiotic that can affect the composition and number of gut microbiota (16-20). In addition, fermentation and production of short chain fatty acids such as acetate, propionate, butyrate, and reduction of lumen PH cause the growth of beneficial bacteria such as Lactobacillus and Bifidbacterium (21). Butyrate is one of the short chain fatty acids produced by fermentation of soluble fiber, and recent studies have shown that it can suppress colon inflammation through inducing apoptosis of T Cells, and via suppressing inflammation by inhibition of Interferon gamma (21).

Dietary fiber seems to improve all the symptoms of patients with IBS such as abdominal discomfort, bloating, digestion and changes in bowel habits, possibly by affecting the gut nervous system, PH changes, and lumen pressure in the intestine and stimulating the release of serotonin hormone, which plays an important role in visceral sensitivity (21). Short chain fatty acids, which are produced by dietary fiber, affect several intestinal hormones such as neuropeptide YY (PYY) and glucagon like peptide. PYY stimulates the absorption of water and electrolytes; in addition to PYY, prostaglandin E2 and vasoactive polypeptide inhibit intestinal stimulation, and this effect can explain the effects of dietary fiber on the gastrointestinal tract and its secretions (22). In a study conducted in Norway, low FODMAP diet altered the density of endocrine glandular cells in the gastrointestinal tract of patients with IBS (23). Since FODMAPs include main sources of dietary fiber, these observations indicate the effect of dietary fiber on endocrine glandular cells. It seems that short-chain fatty acids, especially butyrate-derived fatty acids, affect the neurons of enteric system, whether this effect directly affects the neuromuscular system or involves indirect effects on the glands of gastrointestinal tract is not clear.

One of the strengths of the present study is that it is the first study to investigate the relationship between dietary factors and the risk of IBS. Meanwhile, the confounding variables have been fully identified and entered into the model using other studies, according to the reported cases. Therefore, this study has little residual confounders. However, this issue is not completely ruled out; differences between studies may be due to differences in the assessment of macronutrient intakes, the problem of disposing the pre-and out-of-diet regimen, and the differences in particular foods consumed by each population. 

The present study has several limitations. Although we used a valid and reliable FFQ for assessment of dietary intakes, it is also accompanied by errors in the collection of information that should be considered in interpretation of the findings. There is a possibility of measurement error. In addition, the possibility of selection bias was inevitable in this study. Although randomized sampling was used in this study, it is still possible that samples are not representative of the target population. One of the other limitations in this study was that we did not ask about the stressful life events, as well as the amount of depression and stress from participants, which may affect the onset of the syndrome. Considering the limitations of this study, more comprehensive studies with a higher sample size are recommended to evaluate the nutritional factors associated with IBS.

Our data indicate that higher dietary fiber intake is related to lower risk of IBS. Further prospective studies are recommended to be able to conclude it firmly.

## References

[1] Thompson WG, Longstreth GF, Drossman DA, Heaton KW, Irvine EJ, Muller-Lissner SA (1999). Functional bowel disorders and functional abdominal pain. Gut.

[2] Ritchie J (1973). Pain from distension of the pelvic colon by inflating a balloon in the irritable colon syndrome. Gut.

[3] Spiller RC, Jenkins D, Thornley JP, Hebden JM, Wright T, Skinner M (2000). Increased rectal mucosal enteroendocrine cells, T lymphocytes, and increased gut permeability following acute Campylobacter enteritis and in post-dysenteric irritable bowel syndrome. Gut.

[4] EleonoraDistrutti, Lorenzo Monaldi, Patrizia Ricci, Stefano Fiorucci (2016). Gut microbiota role in irritable bowel syndrome: New therapeutic strategies. World J Gastroenterol.

[5] Zhang Y, Li L, Guo C, Mu D, Feng B, Zuo X (2016). Effects of probiotic type, dose and treatment duration on irritable bowel syndrome diagnosed by Rome III criteria: a meta-analysis. BMC Gastroenterol.

[6] Cuomo R, Andreozzi P, Zito FP, Passananti V, De Carlo G, Sarnelli G (2014). Irritable bowel syndrome and food interaction. World J Gastroenterol.

[7] Halmos EP, Power VA, Shepherd SJ, Gibson PR, Muir JG (2014). A diet low in FODMAPs reduces symptoms of irritable bowel syndrome. Gastroenterology.

[8] Khayyatzadeh SS, Esmaillzadeh A, Saneei P, Keshteli AH, Adibi P (2016). Dietary patterns and prevalence of irritable bowel syndrome in Iranian adults. Neurogastroenterol Motil.

[9] Okami Y, Kato T, Nin G, Harada K, Aoi W, Wada S (2011). Lifestyle and psychological factors related to irritable bowel syndrome in nursing and medical school students. J Gastroenterol.

[10] Chirila I, Petrariu FD, Ciortescu I, Mihai C, Drug VL (2012). Diet and irritable bowel syndrome. J Gastrointestin Liver Dis.

[11] Omagari K, Murayama T, Tanaka Y, Yoshikawa C, Inoue S, Ichimura M (2013). Mental, physical, dietary, and nutritional effects on irritable bowel syndrome in young Japanese women. Intern.

[12] Hayes P, Corish C, O'Mahony E, Quigley EM (2014). A dietary survey of patients with irritable bowel syndrome. J Hum Nutr Diet.

[13] Böhn L, Störsrud S, Simrén M (2013). Nutrient intake in patients with irritable bowel syndrome compared with the general population. Neurogastroenterol Motil.

[14] Böhn L, Störsrud S, Simrén M (2013). Nutrient intake in patients with irritable bowel syndrome compared with the general population. Neurogastroenterol Motil.

[15] Mirmiran P, Esfahani FH, Mehrabi Y, Hedayati M, Azizi F (2010). Reliability and relative validity of an FFQ for nutrients in the Tehran lipid and glucose study. Public Health Nutr.

[16] El-Salhy M, Ystad SO, Mazzawi T, Gundersen D (2017). Dietary fiber in irritable bowel syndrome (Review). Int J Mol Med.

[17] Chutkan R, Fahey G, Wright WL, McRorie J (2012). Viscous versus nonviscous soluble fiber supplements: Mechanisms and evidence for fiber-specific health benefits. J Am Acad Nurse Pract.

[18] McRorie JW, Daggy BP, Morel JG, Diersing PS, Miner PB, Robinson M (1998). Psyllium is superior to docusate sodium for treatment of chronic constipation. Aliment PharmacolTher.

[19] Hekmatdoost A, Wu X, Morampudi V, Innis SM, Jacobson K (2013). Dietary oils modify the host immune response and colonic tissue damage following Citrobacter rodentium infection in mice. Am J Physiol Gastrointest Liver Physiol.

[20] Jalili M, Hekmatdoost A, Vahedi H, Poustchi H, Khademi B, Saadi M (2016). Co-Administration of Soy Isoflavones and Vitamin D in Management of Irritable Bowel Disease. PLoS One.

[21] Vanhoutvin SA, Troost FJ, Kilkens TO, Lindsey PJ, Hamer HM, Jonkers DM (2009). The effects of butyrate enemas on visceral perception in healthy volunteers. Neurogastroenterol Motil.

[22] Sanchez D, Miguel M, Aleixandre A (2012). Dietary fiber, gut peptides, and adipocytokines. J Med Food.

[23] El-Salhy M, Gilja OH, Gundersen D, Hatlebakk JG, Hausken T (2014). Interaction between ingested nutrients and gut endocrine cells in patients with irritable bowel syndrome (review). Int J Mol Med.

